# The RNA shapes studio

**DOI:** 10.1093/bioinformatics/btu649

**Published:** 2014-10-01

**Authors:** Stefan Janssen, Robert Giegerich

**Affiliations:** Practical Computer Science, Faculty of Technology, Bielefeld University, D-33615 Bielefeld, Germany

## Abstract

**Motivation** : Abstract shape analysis, first proposed in 2004, allows one to extract several relevant structures from the folding space of an RNA sequence, preferable to focusing in a single structure of minimal free energy. We report recent extensions to this approach.

**Results** : We have rebuilt the original RNA shapes as a repository of components that allows us to integrate several established tools for RNA structure analysis: RNA shapes , RNA alishapes and pknots RG, including its recent extension p K iss . As a spin-off, we obtain heretofore unavailable functionality: e. g. with p K iss , we can now perform abstract shape analysis for structures holding pseudoknots up to the complexity of kissing hairpin motifs. The new tool p A li K iss can predict kissing hairpin motifs from aligned sequences. Along with the integration, the functionality of the tools was also extended in manifold ways.

**Availability and implementation** : As before, the tool is available on the Bielefeld Bioinformatics server at http://bibiserv.cebitec.uni-bielefeld.de/rnashapesstudio .

**Contact** : bibi-help@cebitec.uni-bielefeld.de

## 1 THE RNA SHAPES STUDIO

### 1.1 Integration of tools for RNA abstract shape analysis

**Fig. 1.**
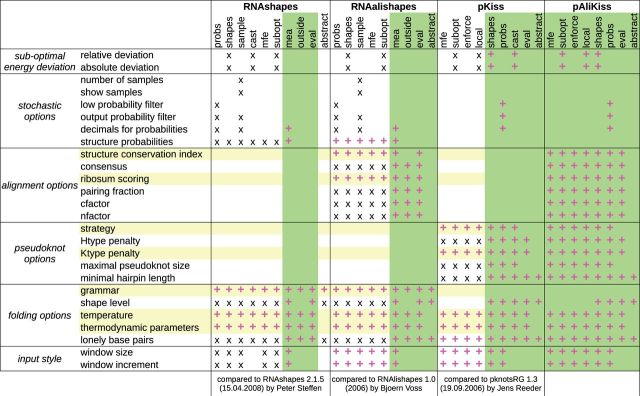
Parameters for the RNA shapes studio. New features are indicated by + . New parameters are highlighted in yellow. New analysis modes are shaded in green

The framework of algebraic dynamic programming (ADP) allows us to express dynamic programming algorithms for sequence analysis on a high level of abstraction. They are composed from signatures, tree grammars and evaluation algebras ( Giegerich *et al.* , 2004a ). Powerful product operations on algebras allow one to derive new types of analysis by the combination of available components, essentially with a single keystroke ( Steffen and Giegerich, 2005 ). Relying on the recent B ellman’s GAP system ( Sauthoff *et al.* , 2013 ), which implements the ADP framework, we have built a repository of components that allows us to integrate several established tools for RNA structure analysis: RNA shapes , RNA alishapes and pknots RG, including its recent extension p K iss . As a spin-off, we obtain heretofore unavailable functionality: e. g. with p K iss , we can now perform abstract shape analysis for structures holding pseudoknots up to the complexity of kissing hairpin motifs. The new tool p A li K iss can predict kissing hairpin motifs from aligned sequences. Along with the integration, the functionality of the tools was also extended in manifold ways. Figure 1 gives an overview.

### 1.2 Integrated tools and their new functionality

#### 1.2.1 Extensions to RNA shapes

It is generally agreed that predicting a single structure of minimal free energy does not adequately capture the subtlety and versatility of RNA structure formation. The RNA shapes tool introduced the notion of abstract shapes ( Giegerich *et al.* , 2004b ; Voß *et al.* , 2006 )—a (mathematically precise) characterization of structures by their arrangement of helices. For example, ‘[[][][]]’ indicates a cloverleaf shape, and ‘[_[_[]_]]’ a single stem-loop with a 5′ bulge and an internal loop. Classical abstract shape analysis reports minimum free energy structures from *different* shape classes, or Boltzmann structure probabilities accumulated by shape. This gives synoptic information about the folding space of a given RNA sequence, without heuristics or sampling. Extending RNA shapes , we added different modes of treating dangling bases (consistent with RNA fold options −d0, −d1 and −d2 ) ( Janssen *et al.* , 2011 ; Lorenz *et al.* , 2011 ), computation of base pair probabilities and maximum expected accuracy (MEA) folding ( Lu *et al.* , 2009 ).

#### 1.2.2 Extensions to RNA alishapes

The work of Voß (2006) combines the ideas of RNA alifold and RNA shapes and performs shape analysis based on pre-aligned RNA sequences. We added the computation of a structure conservation index, different dangling base models, MEA folding and a window mode. RIBOSUM scoring ( Bernhart *et al.* , 2008 ) was added for evaluating sequence similarity.

#### 1.2.3 Extensions to p K iss

In Theis *et al.* (2010) the ideas of pknots RG ( Reeder and Giegerich, 2004 ) are extended to predict (aside from unknotted structures and H-type pseudoknots) RNA structures that exhibit kissing hairpin motifs in an arbitrarily nested fashion, requiring $O(n^{4})$ time and $O(n^{2})$ space. We added shape analysis, probabilities, different folding strategies and different dangling base models. The -cast option provides comparative prediction of pseudoknotted structures as in the RNA cast approach ( Reeder and Giegerich, 2005 ). A window mode was also included.

#### 1.2.4 New tool p A li K iss

The program p A li K iss allows to predict pseudoknots, including kissing hairpins from aligned sequences. Being composed from the grammars and algebras of the other tools, it inherits all the features and options that make sense for it.

#### 1.2.5 Utilities

All tools were augmented with utilities to compute folding energy or abstract shape for sequences that are provided with a structure from an outside source, in a way consistent with the tools’ energy model. The graphical motif description tool L ocomotif ( Reeder *et al.* , 2007 ) now uses modules from the RNA shapes studio. The K not I n F rame ( Theis *et al.* , 2008 ) tool that predicts −1 ribosomal frameshifts has been updated as well.

## 2 APPLICATION CASE: A FRAMESHIFT STIMULATION ELEMENT IN MERS

The Corona virus family contains a frameshift stimulation element ( Baranov *et al.* , 2005 ), where the frameshift is facilitated by a slippery site together with either an H-type or a K-type pseudoknot. R fam ( Burge *et al.* , 2013 ) holds the corresponding family model RF00507, although the tools of R fam cannot explicitly model pseudoknots. p K iss ( Theis *et al.* , 2010 ) in -enforce mode reveals that for 11 family members, minimal free energy structures are H-types, another 11 are K-type pseudoknots and for only one member a purely nested structure has the best energy.

**Fig. 2.**
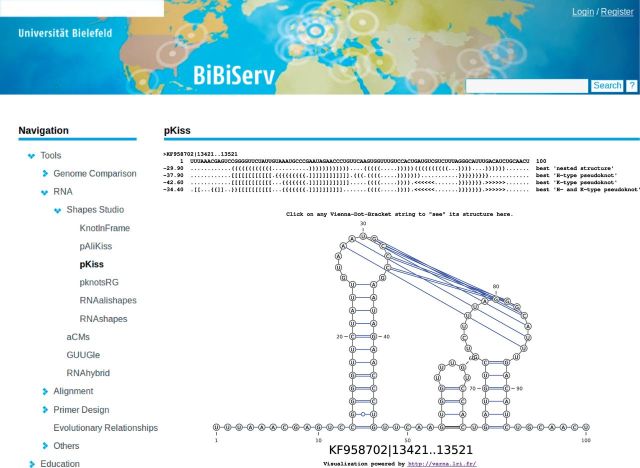
RNA shapes studio result page for folding the MERS example with p K iss . Illustration by VARNA ( Darty *et al.* , 2009 )

The recently sequenced MERS genome (KF958702.1) is annotated with a homologous frameshift site, whereas the structure of the triggering element remains unclear. Structure prediction with p K iss for a 100 bp stretch downstream the slippery site attests a most stable K-type pseudoknot (see Fig. 2 ). A second run of p K iss , this time in probability mode, shows that the shape class of this particular K-type pseudoknot has an overwhelming Boltzmann probability of ≈99% ; leaving not much probability mass for any other shape classes.

## 3 AVAILABILITY

The RNA shapes studio is available at http://bibiserv.cebitec.uni-bielefeld.de/rnashapesstudio . Users can access the B ellman’s GAP source code of all components in the repository, and combine or extend them according to their own goals. This has been done, for example, in Reinkensmeier *et al.* (2011) for defining the CCUCCUCC -motif family in the *Rhizobiales* .

*Conflict of interest* : none declared.
